# Molecular predictive biomarker testing in advanced thyroid cancer – a European consensus

**DOI:** 10.1530/ETJ-25-0024

**Published:** 2025-07-07

**Authors:** Aleš Ryška, Jaume Capdevila, Matthias S Dettmer, Rossella Elisei, Dagmar Führer, Julien Hadoux, Barbara Jarząb, Laura D Locati, Kate Newbold, Giovanni Tallini, Silvia Uccella, Lori Wirth, Ravinder Singh, Iris M Simon, Pilar Camacho, Laura Fugazzola

**Affiliations:** ^1^ The Fingerland Department of Pathology, Charles University, Faculty of Medicine, Hradec Kralove, Czechia; ^2^ University Hospital, Hradec Kralove, Czechia; ^3^ Vall d’Hebron University Hospital, Barcelona, Spain; ^4^ Vall d’Hebron Institute of Oncology (VHIO), Barcelona, Spain; ^5^ Klinikum Stuttgart, Institute of Pathology, Stuttgart, Baden-Württemberg, Germany; ^6^ Institute of Tissue Medicine and Pathology, University of Bern, Bern, Switzerland; ^7^ Endocrine Unit, University Hospital of Pisa, Pisa, Italy; ^8^ Department of Endocrinology, Diabetes and Metabolism, Endocrine Tumour Centre at West German Cancer Centre, University Hospital Essen, University of Duisburg-Essen, Essen, Germany; ^9^ Service d’oncologie endocrinienne, département d’imagerie & ENDOCAN-TUTHYREF Network, Gustave Roussy, Villejuif, France; ^10^ Narodowy Instytut Onkologii im. M. Curie Sklodowskiej, Gliwice Branch, Gliwice, Poland; ^11^ Department of Internal Medicine and Therapeutics, University of Pavia, Pavia, Italy; ^12^ Istituti Clinici Scientifici Maugeri IRCCS, Medical Oncology Unit, Pavia, Italy; ^13^ Royal Marsden NHS Foundation Trust, Thyroid Unit, London, UK; ^14^ University of Bologna, Department of Medical and Surgical Sciences (DIMEC) Bologna, Emilia-Romagna, Italy; ^15^ Humanitas University, Milan, Italy and IRCCS Humanitas Research Hospital, Milan, Italy; ^16^ Department of Medicine, Massachusetts General Hospital, Harvard Medical School, Boston, Massachusetts, USA; ^17^ Eli Lilly and Company, Indianapolis, Indiana, USA; ^18^ Department of Endocrine and Metabolic Diseases, Istituto Auxologico Italiano IRCCS and Department of Pathophysiology and Transplantation, University of Milan, Milan, Italy

**Keywords:** biomarkers, consensus, molecular testing, thyroid cancer, multidisciplinary

## Abstract

As new precision oncology therapies become available in the thyroid cancer (TC) treatment landscape, appropriate and timely biomarker testing is crucial for treatment selection and requires a multidisciplinary approach. Recently published European guidelines on advanced/metastatic TC management include a special focus on biomarker testing. However, to date, there remains a need for comprehensive European guidance for standardized molecular testing strategies in TC that encompass a broad set of targetable or potentially targetable alterations, timing of testing, and patients to be tested. This expert opinion article outlines consensus testing algorithms for differentiated TC, medullary TC, and anaplastic TC from a team of endocrinologists, oncologists, molecular biologists, and pathologists to provide standardized recommendations for physicians involved in treating patients with advanced TC. In the differentiated TC algorithm, patients recommended for comprehensive testing by DNA and RNA next-generation sequencing (NGS) include those whose disease has progressed on or is resistant to radioactive iodine treatment. The medullary TC algorithm recommends RET germline testing for all patients at diagnosis. For patients exhibiting high-risk clinical or pathological features and those whose disease progresses, somatic RET testing with NGS should be discussed and conducted before considering systemic treatment. As anaplastic TC is a highly aggressive disease, molecular reflex testing for *BRAF* mutations is recommended for all patients at diagnosis, followed by DNA and RNA NGS for those who test *BRAF* negative. The article also provides consensus recommendations on the use of tumor tissue for testing and on centralization of molecular testing involving multidisciplinary tumor boards.

## Introduction

Thyroid cancer (TC) incidence has been substantially increasing over the last 30 years, and it holds seventh place in the GLOBOCAN 2022 database for cancer incidence and mortality (1, 2). The three main histotypes of TC include the follicular cell-derived differentiated TC (DTC) and anaplastic TC (ATC), and the C-cell-derived medullary TC (MTC) (3). DTC can be further categorized into papillary TC (PTC, the predominant DTC type), the invasive encapsulated follicular variant of PTC, follicular TC (FTC), oncocytic TC (OTC), and high-grade follicular cell-derived non-anaplastic thyroid carcinoma, including differentiated high-grade thyroid carcinoma and poorly differentiated TC (PDTC) (4, 5, 6). Of note, PTC accounts for 80–85% of diagnosed TC, FTC accounts for 5–15% of TC, and MTC accounts for 5–10% of TC (7, 8, 9). PDTC and OTC comprise approximately 2–5% of all TCs (10). PDTC can occur *de novo* or result from progression from DTC and has an intermediate position between DTC and ATC, reflected in its clinical and pathological features and accounting for the highest mortality rate from non-anaplastic follicular cell-derived TC (11). Oncocytic TC was previously classified as a subtype of FTC but is now considered distinct from FTC based on its different genetic alterations and clinicopathologic features (12).

The cells of DTC retain the ability to accumulate iodine (more in PTC, FTC, and OTC, and less in high-grade differentiated thyroid carcinoma and PDTC), which can be used in the treatment of patients with metastatic DTC (1, 4, 13). Initial treatment of DTC typically consists of either total thyroidectomy or hemithyroidectomy, depending on the size of the malignant tumor, followed by radioactive iodine (RAI) in selected cases with an intermediate risk of recurrence and in all cases with high risk of recurrence. In small DTC, commonly ≤1 cm, active surveillance and/or minimally invasive therapies can be proposed (14, 15). About 5–15% of metastatic DTCs progress to RAI-refractory (RAIR) disease (15, 16). The prognosis for ATC is particularly dismal, as this tumor type is intrinsically highly aggressive and RAIR, with median overall survival of <10 months in historical cohorts (17, 18). Considering MTC, which is RAIR by definition, clinically detected distant metastases are present in approximately 10% of patients at initial diagnosis and may be detected in 18–38% during follow-up (19). Development of structural disease is particularly common in patients with MTC who have documented biochemical incomplete response at the first examination after initial surgical treatment (20). The poor prognosis of RAIR TC and the inefficacy of standard chemotherapy for advanced aggressive TCs prompted the development of novel TC therapies some years ago (16).

Recently, several potentially actionable biomarkers for TC were discovered, allowing for the introduction of targeted therapies in the TC treatment landscape (21, 22). These include B-Raf proto-oncogene (*BRAF*) mutations, rat sarcoma virus (*RAS*) mutations, neurotrophic tyrosine receptor kinase (*NTRK*) gene fusions, and rearranged during transfection (*RET*) fusions and mutations (8, 23, 24). As our understanding of TC biology evolves, the European Society for Medical Oncology (ESMO) scale for clinically actionable molecular targets provides a useful framework for defining clinical evidence-based criteria to prioritize genomic alterations as markers to select patients for targeted therapies (25).

At advanced stages, *BRAF V600E* and *RAS* mutations are frequently present in PTC and are almost always mutually exclusive, representing 40–80% and 5–15% of identified mutations in advanced disease, respectively (Table 1) (9, 26, 27, 28, 29). Most variability in the frequency of *BRAF V600E* mutations is linked to subtypes (e.g., tall cell PTC has very high prevalence of *BRAF V600E*, solid trabecular a relatively high proportion of tyrosine kinase fusions) (9). There are also reports that BRAF mutation frequency might be higher in Asian populations (30). The incidence of *BRAF* and *RAS* mutations in PTC correlates well with the individual histological subtypes: the classical subtype of PTC, as well as other PTC subtypes with a papillary growth pattern, and the infiltrative follicular variant of PTC are classified as *BRAF*-like malignancy, whereas FTC and the invasive encapsulated variant of PTC both belong to the *RAS*-like category (1, 9, 31). In addition, small subsets of PTCs, namely those with papillary growth pattern, may harbor fusions of cancer-related genes, among which *RET*, *NTRK1–3*, *BRAF*, and anaplastic lymphoma kinase (*ALK*) are the most frequent (5). In FTC, *RAS* mutations may be observed in 40–50% of cases, and other common alterations include *PAX8-PPARG* (12–30%) and *PIK3CA* (<10%) (32). The most common molecular alterations in PDTC include *TERT* promoter (40%), *BRAF V600E* (≈25%), and *RAS* (≈25%) mutations (11). In MTC, the molecular hallmark of the familial form is represented by *RET* germline mutations, which are detected in 95% of cases, whereas somatic *RET* mutations may be observed in 25–40% of sporadic MTC (33). *RET* fusions are mainly detected in PTCs (10%) (8, 34), particularly in younger patients.

**Table 1:** Overview of common molecular alterations in TC.

DTC			MTC (8, 33, 34)	ATC (9)
Well differentiated TC		PDTC (11)		
FTC (32)	PTC (8, 9, 26, 27, 28, 29, 34)			
*RAS* (40–50%)	*BRAF* V600E (40–80%)	*TERT* (40%)	Germline *RET* (95%)	*TP53* (40–80%)
*PIK3CA* (<10%)	*RAS* (5–15%)	*BRAF V600E* (≈25%)	Somatic *RET* (25–40%)	*TERT* (30–75%)
Gene fusions mainly *PAX8-PPARG* (12–30%)	Gene fusions include *RET (10%), NTRK1–3, BRAF*, *ALK*	*RAS* (≈25%)		*RAS* (10–50%)
				*BRAF* V600E (10–50%)
				*PIK3CA* (5–25%)
				*PTEN* (10–15%)
				*EIF1AX* (5–15%)
				Gene fusions mainly *RET*, others (0–5%)

*ALK*, anaplastic lymphoma kinase; ATC, anaplastic thyroid cancer; *BRAF, *B-Raf proto-oncogene; DTC, differentiated thyroid cancer; *EIF1AX, *eukaryotic translation initiation factor 1A, X-chromosomal; FTC, follicular thyroid cancer; MTC, medullary thyroid cancer; *NTRK, *neurotrophic tyrosine receptor kinase; *PAX8-PPARG,* paired box 8-peroxisome proliferator-activated receptor gamma; PDTC, poorly differentiated thyroid cancer; *PIK3CA,* phosphatidylinositol 3 kinase; PTC, papillary thyroid cancer; *PTEN, *phosphatase and tensin homolog deleted on chromosome 10; *RAS,* rat sarcoma virus; *RET,* rearranged during transfection; *TERT, *telomerase reverse transcriptase; *TP53, *tumor protein 53; TC, thyroid cancer.

ATC might represent a dedifferentiation of a DTC and tends to retain the molecular alterations of the original differentiated component, which is why routine molecular profiling at time of diagnosis of ATC is recommended (35). Common mutations in ATC include *TP53* (40–80%), *TERT* (30–75%), *RAS* (10–50%), and *BRAF V600E* (10–50%) (Table 1) (9).

Available treatment options for gene-alteration-driven metastatic RAIR TC include selective RET inhibitors such as selpercatinib (and pralsetinib in the USA), NTRK inhibitors entrectinib and larotrectinib (and repotrectinib in the USA), and BRAF inhibitors such as dabrafenib and vemurafenib. The latter inhibitors may be given in combination with mitogen-activated protein kinase (MEK) inhibitors, although data supporting this combination are limited (36, 37). *RAS* mutations are potentially actionable targets. Although no RAS inhibitors are yet approved for patients with advanced TC, data are emerging in other solid tumors (38).

The 5th edition of the World Health Organization Classification of Endocrine and Neuroendocrine Tumors recognizes the importance of classifying tumors based on both histopathological features and genomic alterations, reflecting our current understanding of tumor pathogenesis and biology and facilitating the early selection of appropriate treatment options, including molecular targeted therapies (5, 39). For optimal therapeutic management of individual patients, both accurate tumor classification and correct disease staging are required, as well as timely testing of TC for potentially targetable genomic alterations in cases where there are clinically relevant management consequences. A published US-based expert consensus presented preliminary guidelines for testing of genomic alterations in TC, including DTC, MTC, and ATC (40). In addition, ESMO recommendations for actionable genomic alterations in advanced TC, including *RET* alteration-driven cancer and cancers with *NTRK* fusions, and next-generation sequencing (NGS), are in place to support guidelines for TC testing and treatment (41, 42, 43), but do not define the ideal timing for TC testing. In this context, MTC requires particular attention for two reasons. First, a significant subgroup of patients with MTC shows familial background, so germline testing to identify/exclude hereditary process is needed; second, it is the tumor type for which RAI is not a therapeutic option. In principle, all patients with progressive and advanced metastatic MTC are potential candidates for alternative systemic treatment, and targeted treatment is an approach with proven efficacy and safety in patients with aggressive metastatic disease (44). Efforts have been undertaken to generate testing guidelines and recommendations for MTC and PTC (8, 40, 45). In addition, algorithm proposals for molecular diagnostics of predictive biomarkers in sporadic MTC and follicular cell-derived thyroid malignancies that are RAIR were previously published focusing on a single or multiple biomarkers (22, 46, 47, 48). European recommendations regarding advanced/metastatic TC management have recently been reviewed with a special focus on molecular testing and inclusion of comparative findings with real-world practice (45). Nevertheless, to date, there remains a need for comprehensive European recommendations for standardized molecular testing strategies in TC encompassing a broad set of targetable or potentially targetable alterations, timing of testing, patients to be tested, and treatment decisions. Therefore, improved guidance is needed (49).

This article aims to present the expert consensus of a team of endocrinologists, oncologists, molecular biologists, and pathologists on a comprehensive TC testing algorithm. Specifically, a practical and pragmatic algorithm encompassing clinical factors and testing optimization is proposed for patients with DTC, MTC, or ATC throughout their treatment journey from the point of diagnosis. This will help to ensure that all patients with TC are tested and treated according to best clinical practices. Rapidly evolving technological advances, along with cost and reimbursement issues, will necessitate regular updates to ensure standardization of molecular predictive biomarker testing in advanced TC and implementation of best-evidence practice. Consideration is given to identifying biomarkers that are targetable by approved drugs for TC or approved drugs in other indications (thereby providing indirect evidence of efficacy, e.g. *RAS* mutations), as well as prognostic markers yet to have an impact on clinical management (e.g. *TERT* mutations).

## Consensus DTC testing algorithm

Upon diagnosis of DTC, patients with high risk of recurrence and selected cases with intermediate risk of recurrence usually undergo thyroidectomy, followed by RAI treatment when appropriate. For most patients, disease response is positive, with no signs of progression. However, for a small fraction of patients, DTC becomes RAIR, leading to disease progression. A small number of patients might also be identified with advanced disease at initial diagnosis. Molecular testing for all advanced DTC with signs of progression (or non-stable disease) is recommended, although the possibility of finding a driver mutation will vary depending on the subcategory of DTC (e.g. PTC, FTC, or OTC) (50, 51). Ideally, a biopsy is taken at the time of progression and analyzed by DNA and RNA NGS. In cases where a biopsy is not feasible, an alternative would be to obtain a nucleic acid sample from the initial tissue sample (see section on Consensus recommendation on the use of tumor tissue for testing).

If comprehensive NGS testing of all patients with advanced DTC is not an option, a two-step testing approach can be used (22, 46). The first step is to conduct tests for *BRAF*, the most common actionable mutation in DTC. *BRAF* mutations can be detected by immunohistochemistry (IHC), although this may not be sufficient for the approved use of BRAF inhibitors in some countries; therefore, detection by polymerase chain reaction (PCR) and direct sequencing is preferable, and IHC may be used for triaging cases to be sent for molecular analysis. *TERT* mutations are also frequent in advanced TC, in association with either *BRAF* or *RAS* mutations, and can be tested mainly for prognostic purposes (52). Patients with *BRAF* and *RAS* wild-type status can be tested by RNA NGS to identify *NTRK* and *RET* fusions that allow treatment with specific kinase inhibitors, or to identify *ALK* or *ROS1* fusions or other rare genomic alterations that might allow patients to be included in clinical trials. A comprehensive analysis of DNA by NGS can give further insights into less common mutations. If molecular testing is unavailable at the local hospital, appropriate hub centers should be used (i.e. dedicated, accredited laboratories with experience in testing biomarkers).

**Figure 1:**
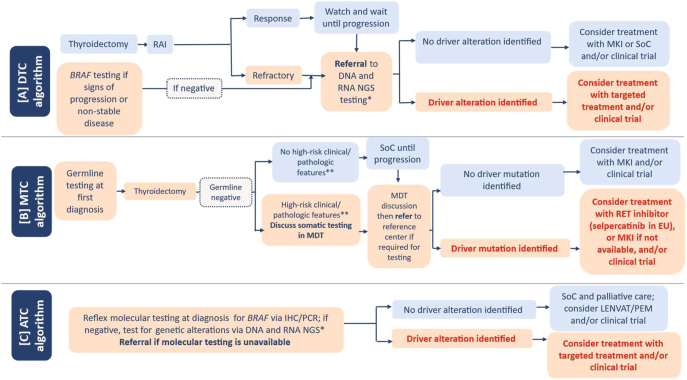
Expert consensus testing algorithm for (A) differentiated thyroid cancer (DTC), (B) medullary thyroid cancer (MTC), and (C) anaplastic thyroid cancer (ATC). *If next-generation sequencing (NGS) testing and referral are not feasible, single-gene analysis of druggable genetic alterations is recommended; **Examples of high-risk clinical or pathologic features: high calcitonin levels both before and after surgery, short calcitonin doubling time, early distant metastases, histologically high-grade disease, tumor burden, and disease stage. IHC, immunohistochemistry; LENVAT/PEM, lenvatinib plus pembrolizumab; MDT, multidisciplinary teams; MKI, multikinase inhibitor; PCR, polymerase chain reaction; RAI, radioactive iodine; SoC, standard of care. Boxes in light red outline an algorithm path that involves testing and treatment with driver mutations. Boxes in light blue indicate steps in the algorithm that do not require testing.

Comprehensive DNA- and RNA-based NGS testing, including tumor mutational burden (TMB) where available is strongly recommended for patients with RAIR and progressive disease. Patients with unresectable disease should undergo testing because a neoadjuvant treatment can be considered in some cases. Targeted treatment with specific kinase inhibitors should be considered if a driver gene alteration is identified in patients whose disease is RAIR or has progressed; if no driver alteration is identified, the authors recommend treatment with multikinase inhibitors (MKIs) or the standard of care (SoC). Inclusion into clinical trials is also an option, regardless of whether a genomic alteration is identified. A summary of the consensus DTC testing algorithm is presented in Fig. 1A.

## Consensus MTC testing algorithm

Germline *RET*-mutation testing is recommended for all patients with MTC at diagnosis. This consensus aligns with MTC clinical practice guidelines from the National Comprehensive Cancer Network (53) and previous recommendations from European associations (54, 55). The consensus also recommends performing germline testing in high-volume, specialized genetic laboratories. In patients with germline wild-type *RET* status, tumor tissue should be tested for somatic *RET* mutations at the time of diagnosis only if they present with high-risk clinical or pathological features. Those without high-risk features should proceed to be treated with the SoC. Patients who harbor germline *RET* mutations do not require somatic testing for *RET* mutations. Experts agree that high-risk clinical or pathological features include high calcitonin levels both before and after surgery, short calcitonin doubling time, early distant metastases, and histologically high-grade disease. For example, ESMO clinical practice guidelines note that detectable calcitonin and abnormal carcinoembryonic antigen (CEA) after surgery indicates a biochemical incomplete response and would mandate calcitonin and CEA every 3–6 months to determine doubling time; doubling time less than 24 months is associated with progressive disease (54). Currently, results from patient-based studies provide recommendations for grading of MTCs based on mitotic count, Ki67 proliferative index, tumor necrosis, lymphovascular invasion, and local/distant metastases (56, 57). Again, appropriate test centers should be used for patients with high-risk clinical or pathological features.

This consensus recommends somatic *RET*-mutation testing for all patients with MTC whose disease has progressed and who either have a wild-type result from *RET* germline testing or the germline test result is not available. The recommended somatic testing method is NGS, aligning with the current ESMO recommendations for the detection of *RET* fusions and mutations (41). Most NGS panels can detect all therapeutically relevant genomic alterations. Of note, if NGS is not available, testing can be performed by single-gene methods such as PCR and Sanger sequencing, as the method can detect *RET* hotspot mutations. Somatic testing should always be discussed and conducted with a multidisciplinary approach after the treating physician has documented progression or clinical high-risk features, or the pathologist has identified high-risk features such as a high mitotic count or tumor necrosis (56). The consensus also recommends that if the primary tumor harbors a *RAS* mutation, *RET*-mutation testing is not necessary, at least at initial diagnosis, since these mutations are mutually exclusive.

If a *RET* mutation is found, either germline or somatic, in patients with aggressive metastatic disease, treatments with RET inhibitors (in the EU, the selective RET inhibitor selpercatinib) or the MKI vandetanib are available. In any case, and particularly if no driver or targetable mutations are identified, treatment with the MKI cabozantinib can be offered, too. The indication for systemic treatment and type of medication should always be based on a multidisciplinary tumor board (MDTB) decision.

The consensus MTC testing algorithm is presented in Fig. 1B. Specific recommendation statements are presented in Table 2.

**Table 2:** Table outlining expert recommendations for TC testing.*

**(A) DTC testing algorithm**	
1	DNA and RNA biomarker testing is recommended for all patients with advanced RAIR progressive disease. If it is not possible to test with both, start with RNA testing
2	Referral to specialized centers for molecular testing is recommended if not available locally
3	Single-gene *BRAF* testing can be performed to identify patients with *BRAF* wild-type status, who then should receive DNA and RNA NGS analysis (to identify biomarker fusions)
4	If no driver alteration is identified upon RNA NGS testing, DNA NGS and/or a more comprehensive NGS test is recommended
5	If a driver alteration is identified upon DNA/RNA NGS testing, targeted treatment (e.g., kinase inhibitors) may be considered when clinically indicated
6	Consider inclusion into clinical trials
**(B) MTC testing algorithm**	
1	Germline *RET*-mutation testing is recommended at first diagnosis
2	Patients with a negative *RET*-germline test exhibiting high-risk clinical or pathological features, and patients whose disease progresses, should be discussed in an MDTB to determine the need for somatic *RET*-mutation testing
3	If no driver mutation was identified after somatic *RET*-mutation testing, cabozantinib is approved in the EU for treatment in indicated cases
4	If a *RET* mutation is identified, treatment with a selective RET inhibitor (in the EU, selpercatinib) may be considered vs vandetanib/cabozantinib (both are approved in the EU); the decision on initiation and type of treatment should be made in an MDTB
5	Consider inclusion into clinical trials
**(C) ATC testing algorithm**	
1	Urgent reflex molecular testing for *BRAF* mutations is recommended at diagnosis (in patients with limited tissue availability, NGS is preferable). If negative for *BRAF* mutations, DNA and RNA NGS testing is recommended. Immediate contact with, and ideally referral to, expert centers is recommended
2	If no driver alteration is identified after testing, SoC and palliative care are recommended; lenvatinib plus pembrolizumab may also be considered
3	If a driver alteration is identified after testing, targeted treatment (e.g., specific kinase inhibitors) may be considered
4	Consider inclusion into clinical trials

* If NGS testing and referral are not feasible, single-gene analysis of druggable genetic alterations is recommended.

ATC, anaplastic thyroid cancer; DTC, differentiated thyroid cancer; MDTB, multidisciplinary tumor boards; MKI, multikinase inhibitor; MTC, medullary thyroid cancer; NGS, next-generation sequencing; RAIR, radioactive iodine refractory; SoC, standard of care; TC, thyroid cancer.

## Consensus ATC testing algorithm

In concordance with previously published recommendations for ATC (40, 46), this expert group strongly supports reflex urgent/immediate molecular testing for all patients with ATC at diagnosis. The recommended methods are PCR assay and mutation-specific IHC for *BRAF* V600E, which can detect these mutations within a short turnaround period (40), as well as large-panel DNA- and RNA-based NGS to identify all therapeutically relevant common and rare alterations in potentially actionable biomarkers. A core biopsy (or even fine-needle aspiration (FNA) with sufficient cells) can be used for molecular analysis, especially in patients who are not candidates for surgery. In many cases, patients with ATC will be treated in centers where molecular testing is available; if not, then standard referral to such a center is strongly recommended to enable access to clinical trials and the latest treatment advances. When driver gene alterations are identified, targeted treatment should be considered, possibly within clinical trials, especially if approved drugs are not available. For example, combined use of BRAF inhibitors and MKIs is approved in the USA but not in the EU for patients with ATC harboring *BRAF* mutations. In the absence of driver alterations, treatment and management could include MKI therapy (possibly with a checkpoint inhibitor and within a clinical trial to evaluate efficacy and safety), or other appropriate SoC and palliative care options. As noted, TMB may also provide clinically relevant information, as, for example, a checkpoint inhibitor is approved in the USA (but not yet in the EU) for solid tumors with elevated TMB. Testing for programmed cell death ligand 1 may also be considered for patients with advanced TC, such as ATC or PDTC, although a definitive recommendation is not feasible because the clinical efficacy data for checkpoint inhibitor therapy are limited (58, 59). A summary of the recommended ATC algorithm is presented in Fig. 1C. Recommendation statements are presented in Table 2.

## Consensus recommendation on the use of tumor tissue for testing

The current recommendation emphasizes the preference for using the most recent specimen from tumor tissue for molecular testing. It can be taken from either the primary tumor or the most accessible metastatic site, even if some tumor deposits (e.g., bone lesion) carry some technical challenges for NGS. If such material is not available or sampling of recurring/metastasizing tumor is not feasible, molecular testing of archived primary tumor tissue can be acceptable if the formalin-fixed, paraffin-embedded material was handled and preserved appropriately (60), because DNA in such tissue blocks is stable for many years or even decades, whereas RNA is less stable, particularly if the fixation or tissue block storage is suboptimal. The main limitation of analysis of initial tissue samples taken before the treatment is in the potential of missing new mutations acquired during disease progression. The consensus also notes that samples eligible for testing can be obtained by FNA.

General considerations relating to tissue quality and tissue processing, especially biopsy samples, are well described (61, 62). Although these sources define the optimal use of lung cancer material, the recommendations are also fully applicable to other tissues. Sample storage is a crucial aspect in any cancer diagnostics and treatment. Therefore, the expert consensus recommends a tracking system to store, locate, and test tissue samples at the appropriate times. The importance of sample storage was previously highlighted in a publication focused on a holistic recommendation in lung cancer diagnostics for optimal results (63). Moreover, re-biopsies are recommended if the stored samples are not fit for diagnostic tests. Although the use of circulating tumor DNA has not been widely studied as a biomarker in advanced TC, preliminary data suggest it may have clinical applicability, especially for cases with high tumor burden (64, 65), taking into account the limitations of the sensitivity and specificity of this approach.

## Consensus recommendation on molecular testing centralization and involvement of a multidisciplinary team in molecular tumor boards (MTBs)

The expert consensus concluded that optimal treatment of patients with advanced TC can only be provided with a multidisciplinary approach, where endocrinologists, nuclear medicine specialists, oncologists, pathologists, radiotherapists, surgeons, nurses, and other healthcare experts can discuss each individual patient case. The expert consensus recommends the establishment of a referral system for molecular testing and treatment of patients with advanced TC, with an emphasis on the importance of support from specialized institutions for smaller centers.

Despite the recommendation of the ESMO Precision Medicine Working Group in 2024 to provide multigene sequencing to patients with TC (42), in the real world, the practice of molecular testing is, in general, still rare and very heterogeneous among European countries (66, 67). Results of a survey involving TC experts from 18 European countries showed that, among respondents who regularly prescribed molecular genotyping (*n* = 38), the preferred genotyping methods were DNA-based techniques for gene mutations (92%) and RNA-based techniques for gene fusions (68%); the main source of testing reimbursement was by national healthcare systems (74%) (68). Among those who were routinely involved in managing aggressive TC but did not prescribe molecular analysis (*n* = 9), the main reasons were lack of reimbursement (47%), access to a laboratory facility performing these tests (47%), and lack of access to targeted therapies (40%). These latter respondents were clustered within Bulgaria, Greece, Lithuania, Poland, and the Republic of North Macedonia (68). An analysis of the availability, quality, and reimbursement of biomarker testing across Europe showed that countries in Northern and Western Europe generally perform well in biomarker testing, reflecting their higher investment in healthcare, whereas Baltic countries and those in Southern and Central Europe have more variability in access and funding, and Eastern European countries require more significant structural changes to achieve equitable access to quality biomarker testing (69). When resources and/or access to multigene sequencing are limited, prioritization strategies for specific biomarkers could be considered, such as testing algorithms proposed elsewhere (22, 46).

Although cost-benefit analysis showed that testing is cost-effective if more than two or three alterations are actionable (70), high-volume testing is required to reach optimal cost-effectiveness of comprehensive NGS testing (66). Only centralized molecular testing facilities within a region, province, or metropolitan area allow for referred, comprehensive, and predictive biomarker testing for all patients to enable optimal utilization of equipment and personnel (66). High volumes of samples tested in such laboratories also enable a short turnaround time for the test, which is crucial, particularly for patients with rapidly progressing disease.

A care pathway with a regional network has proven successful in the Netherlands, with a decrease in second-opinion referrals of patients to a designated academic hospital, while maintaining referrals for tertiary care (71). Furthermore, the uniform care pathway and regional MDTB were highly valued among participating specialists. Therefore, the authors recommend referring patients with advanced TC to specialized referral centers. These dedicated centers host MTBs to discuss and provide treatment recommendations for patients with complex or rare molecular test results. Using a centralized approach, referral centers can develop and maintain the required expertise in molecular diagnostics and targeted treatment, and provide access to clinical trials when appropriate. For example, ENDOCAN-TUTHYREF is a national network in France dedicated to the improved management of refractory TC using a network of medical centers specializing in the management of TC, along with various other means such as regional consultation meetings, educational opportunities, and a national database (72).

## Challenges and future perspectives

This expert recommendation for testing in TC highlights the appropriate tests to be undertaken at specific stages within the patient’s journey in the disease. Although these recommendations are provided by mostly European experts, they can serve as a framework for other regions to encourage optimal testing and care globally for patients with TC.

Molecular predictive biomarker testing and treatment in advanced TC involve many challenges. Among these are the costs and accessibility of molecular tests and treatment. NGS is not reimbursed in most countries, especially for TC, although costs related to NGS account for only a small proportion of the overall costs of TC management. In a recent analysis, the cost per correctly identified patient (CCIP) was compared between sequential single-gene testing (SGT) versus multiplex NGS in different tumor types using a genomic testing cost calculator developed based on clinically actionable genomic alterations identified in the ESMO Scale for Clinical Actionability of molecular Targets (70). Although TC was not considered in the analysis, results showed that CCIP was lower for NGS than sequential SGT in most cancer types evaluated. This supports the case for using NGS over SGT, which could benefit patients through early detection of potentially targetable biomarkers and lead to improved clinical outcomes while offsetting the costs of sequential SGTs (70). As highlighted in an ESMO study on accessibility of biomolecular technologies in Europe, a key barrier to accessing NGS is reimbursement (67). The study concluded that addressing limited access to advanced biomolecular technologies, and as a consequence to innovative anticancer strategies, is an important step to reduce inequalities in the era of precision medicine (67). This was echoed by an expert European panel recommending improved infrastructure and funding, as well as multi-stakeholder collaboration between national and European initiatives, to reinforce efforts to improve TC patient care (73). Others note that it is unhelpful if a precision medicine is reimbursed but the cost of testing is not, and suggest that health technology assessment bodies should evaluate test–treatment combinations within cost-effectiveness analyses (74).

As noted earlier, some treatments for advanced TC are approved in the USA but not in the EU, and reimbursement may vary between European countries for drugs approved by the European Medicines Agency (e.g. cabozantinib is approved but not reimbursed for MTC in Spain). Other important challenges include improving awareness of current clinically relevant data among specialists and other physicians involved in the treatment of patients with advanced TC; defining optimal timing of molecular predictive biomarker testing; determining the impact of treatment decisions based on available data, including the effect on overall survival; establishing appropriate sequential treatment strategies; and ensuring correct sampling and molecular biomarker testing and appropriate interpretation of results. Initiatives such as the ENDOCAN-TUTHYREF network can provide potential solutions to meet these challenges, and similar initiatives could be considered in other geographic areas.

In current practice, timely referral to specialized centers or access to appropriate testing facilities when molecular testing is unavailable is lacking. Delays in appropriate testing may affect treatment and lead to a less favorable outcome. Moreover, the involvement of endocrinologists, oncologists, and pathologists within the MTB is vital to ensure appropriate expertise, to prevent misinterpretation of test results or loss of information, and to guarantee optimal testing and treatment decisions, emphasizing the importance of collaboration between disciplines in TC.

Throughout the patient’s journey, different types of testing are conducted depending on disease characteristics and status. For example, for patients with MTC, somatic testing follows germline testing if the germline results are negative. Therefore, there is a need for enhanced communication between clinical geneticists (providing germline testing) and pathologists (responsible for somatic testing) within or between the centers to direct optimal testing for germline-negative patients with MTC.

This article presents expert consensus on testing guidelines for patients with DTC, MTC, and ATC. Overall, a holistic algorithm is presented encompassing testing and treatment decisions throughout the patient’s journey. This algorithm also emphasizes the need for multidisciplinary collaborations, in addition to a well-established diagnostics network. Moreover, this algorithm can be employed by MTBs to inform testing and treatment decisions. To improve on current practice and provide optimal TC biomarker testing strategies, the consensus testing guidelines both focus on biomarkers that are targetable by approved drugs for TC and consider biomarkers targetable by drugs approved for other indications, as well as predictive markers yet to have an impact on clinical management. Other useful resources for molecular predictive biomarker testing in advanced TC in Europe include the European Reference Network on rare endocrine conditions (Endo-ERN) and EURACAN, the European Reference Network for all rare adult solid cancer.

## Declaration of interest

AR has participated at advisory board meetings and/or as an invited speaker for Amgen, AstraZeneca, Bayer, BMS, Eli Lilly, Gilead, Merck, MSD, Pfizer, Roche, and Sanofi, and has received travel support from Gilead and Sanofi. JC has served as advisor and/or speaker for and/or received research support from Advanced Accelerator Applications, Advanz, Amgen, AstraZeneca, Bayer, Eisai, Eli Lilly, Esteve, Exelixis, Gilead, Hudchmed, Incyte, Ipsen, ITM, Merck Serono, Novartis, Pfizer, Roche, and Sanofi. MSD declares no competing interests. RE is a consultant for Bayer, Eisai, Eli Lilly, IPSEN, and Menarini. DF declares no competing interests. JH has served as advisor and/or speaker for and/or received funding for continuing medical education and/or research grants from AAA, Bayer, Eisai, Eli Lilly, HRA Pharma, IPSEN, ITM, Novartis, Pharma Mar, Roche, and Sanofi. BJ declares consultancy for AstraZeneca and employment by AstraZeneca, Bayer, Eisai, Exelixis, Novartis, OxiGene, Pfizer, and Roche for clinical trials, and has received payment for lecturing and developing educational presentations and/or received travel/meeting expenses from Ipsen, Novartis, and Sanofi. BJ is also a coauthor of patent application EP22460030, which is intended to grant an EPO patent. LDL has participated in advisory board meetings for and/or received conference honoraria from Bayer, Eisai, Eli Lilly, IPSEN, Istituto Gentili Srl, Janssen-Cilag, Merck Serono, MSD, New Bridge, Novartis, Roche, Sanofi, Seagen, and Sunpharma, and has received travel support from Gilead. KN has served on the speakers bureau for Eisai. GT declares no competing interests. SU declares no competing interests. LW has received honoraria for advisory roles from Bayer, Blueprint Medicines, Coherus, Eisai, Eli Lilly, Ellipses, EMD Serono, Exelixis, Illumina, Merck, Nested, Novartis, and Tubulis, and received honoraria for serving on a data safety monitoring board from PDS Biotechnology Corporation. RS, IMS, and PC are employees of Eli Lilly. LF has served as a consultant for Eisai, Eli Lilly, and Ipsen.

## Funding

Funding for the development of this manuscript was provided by Eli Lilly and Company.

## Author contribution statement

All authors contributed to interpretation of data, development of consensus recommendations, and drafting the manuscript. All authors read and approved the final submitted version.
